# Blunt trauma pancreatic duct injury managed by non-operative technique, a case study and literature review^☆^

**DOI:** 10.1016/j.tcr.2015.03.003

**Published:** 2015-06-16

**Authors:** A. Zala, R. Gaszynski, G. Punch

**Affiliations:** a Nepean Hospital, Derby St., Kingswood 2747, NSW, Australia; b Liverpool Hospital, Elizabeth St., Liverpool 2170, NSW, Australia

**Keywords:** Trauma, Blunt injury, Pancreas, Non-operative

## Abstract

We describe the case of a 15 year old boy who presented with generalised abdominal pain following a seemingly minor collision at weekend soccer. Investigation revealed a grade IV pancreatic injury that was subsequently managed with pancreatic stent insertion by endoscopic retrograde cholangiopancreatography (ERCP) and total parenteral nutrition (TPN) prior to recommencing low fat diet 10 days post-injury.

## Case report

A previously well 15 year old male presented to the Emergency department at 1600 h, 4 hours after a seemingly minor blunt abdominal injury during a weekend soccer match. Initially following the impact of another player's knee to the abdomen, the adolescent rested out of the game for half an hour before returning to play the second half. Following the game, he returned home, tolerated an afternoon meal before the gradual onset of constant, dull, severe, non-radiating, generalised abdominal pain that was not exacerbated by movement. Due to family concerns, the patient presented to our Level I Tertiary referral trauma centre by own means. He was normotensive and not tachycardic. The patient had no significant medical history. Physical examination revealed a guarded abdomen. Focused Assessment with Sonography for Trauma (FAST) suggested a trace of free fluid in the sagittal pelvic image. The initial blood pathology was normal except for an elevation of amylase 410 units/l, lipase 771 units/l and white cell count 12.2 × 10^9^/l. He was admitted for observation and serial clinical and FAST assessments.

**Fig. 1:**
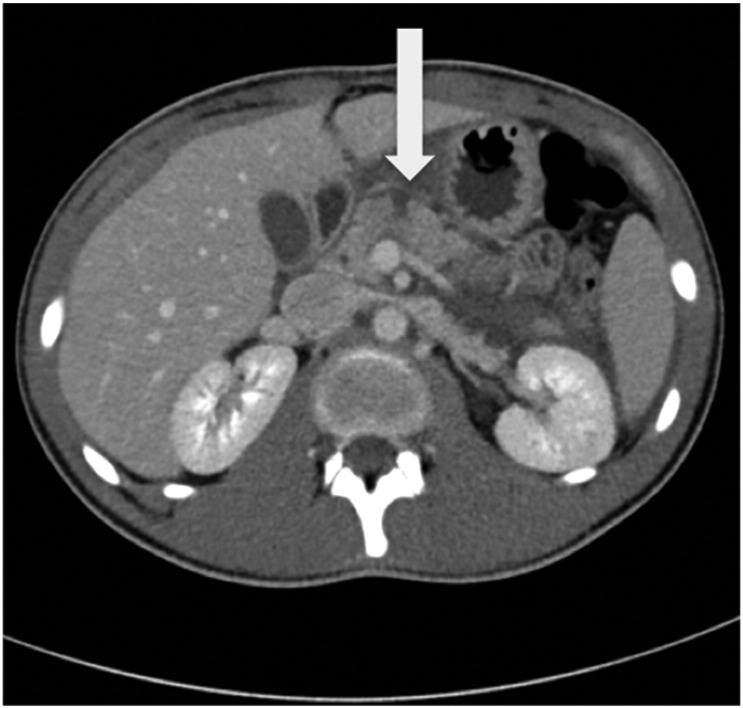
Initial abdominal CT 24 h post-injury showing transection of the neck of the pancreas consistent with AAST-OIS Grade IV injury.

The patient's symptoms failed to improve after 24 h observation, during which time he developed shoulder tip and central back pain. A repeat FAST 24 h post-admission again revealed possible trace free fluid in the sagittal pelvic images with new concerns regarding trace free fluid to the spleno-renal angle. A computed-tomography (CT) scan was performed with intravenous contrast. This revealed a transverse laceration through the neck of the pancreas with normal enhancement of the pancreas and no pancreatic duct dilatation (Fig. 1) consistent with American Association for the Surgery of Trauma (AAST) Organ Injury Scale (OIS) grade IV injury. Additionally there was a small volume, low density intraperitoneal fluid, retroperitoneal stranding, omental contusion and a small linear laceration of the posterior left kidney without peri-nephric collection (AAST-OIS Grade II injury). There was no injury to the other solid organs or the duodenum.

**Fig. 2:**
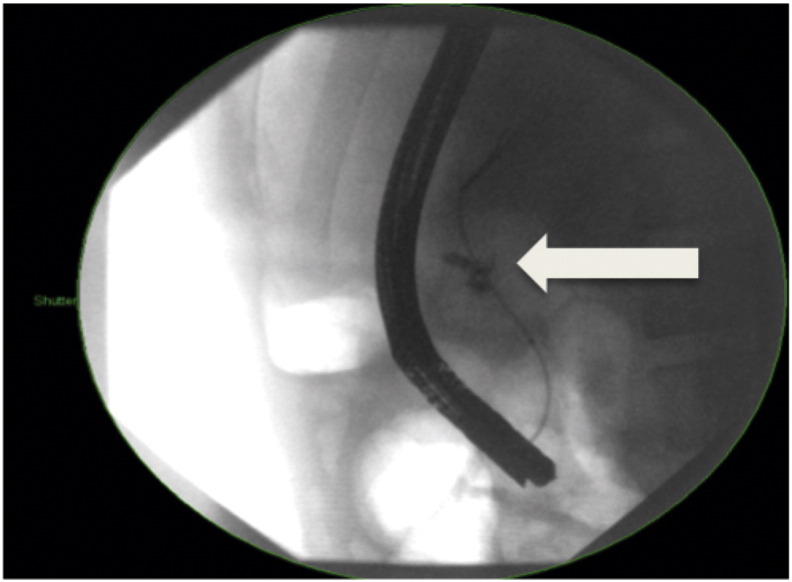
Initial ERCP showing contrast extravasation from the main pancreatic duct and the neck of the pancreas, managed with pancreatic duct stent.

**Fig. 3:**
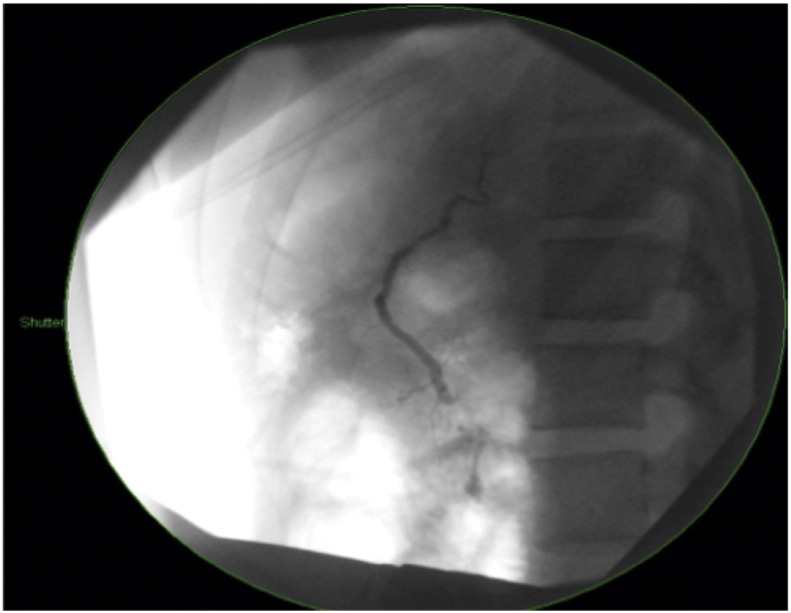
Serial ERCP 4 weeks post-injury showing patent and healed main pancreatic duct.

The patient was kept nil by mouth with intravenous antibiotics, TPN and octreotide 100 mg three times a day via a percutaneous inserted central catheter (PICC). Ongoing central abdominal pain consistent with acute pancreatitis prompted further imaging on day 2 by magnetic resonance cholangiopancreatography (MRCP). This confirmed the CT findings and the patient was booked for ERCP the following day. At ERCP, the pancreatic duct was selectively cannulated and contrast extravasation was seen at the pancreatic neck (Fig. 2). A 5 cm 5Fr pigtail plastic stent was inserted across the defect and the patient remained on TPN and octreotide for 10 days. Repeat MRCP 10 days later showed a contiguous main pancreatic duct of normal calibre and appearance with no evidence of transection and improvement of the peri-pancreatic oedema. The patient was allowed to resume a low fat diet and TPN was weaned and ceased. The patient returned home on pantoprazole 40 mg daily, he was electively brought back to hospital 12 days later for ERCP and stent removal. At ERCP the pigtail stent was not seen, having likely passed out, and there was no evidence of extravasation of contrast, consistent with resolution of injury (Fig. 3). Follow-up as an outpatient was without incident over a three-month period. The patient has been advised not to partake in contact sports for six months.

## Case discussion

Injury to the pancreas is rare in adolescent trauma, with the most common aetiology being motor vehicle accident followed by domestic violence and bicycle crashes [1]. Pancreatic injury is a common diagnostic dilemma following blunt abdominal trauma as symptoms are often insidious in onset and enzyme markers (amylase and lipase) are known not to correlate with severity of injury, as well as being normal in a proportion of injuries [2], [12]. Early diagnosis is crucial in pancreatic injury and whilst CT and MRCP are good non-invasive imaging modalities ERCP is proven to be the most specific and sensitive diagnostic tool [3], [4], [5].

Management of pancreatic injury is based on two factors: integrity of the main pancreatic duct and the location of the pancreatic injury, both of which constitute the AAST-OIS grading system. The evolution in management of blunt pancreatic injuries over the last 20 years has trended towards non-operative management of lower grade injuries with surgical intervention reserved for high grade injuries [2]. The decision for conservative over operative intervention depends on the physiologic stability of the patient, whether the injury is isolated and suitability of the injury for endoscopic treatment [6], [7], [8].

Current literature is divided on the benefits of operative intervention with outcome measures focused on length of hospital stay, failure of non-operative management, need for repeat intervention and rates of surgical complications [3], [10], [11]. There are no Australian based trauma consensus guidelines but non-operative management is usually advocated in the first instance. The most common complication is an increased rate of pseudocyst occurrence, especially in Grade II and Grade III injuries [9]. Most pseudocysts can be managed conservatively, with very little effect on long term endocrine and exocrine function [13]. Other complications include pancreatic fistula, sepsis and recurrent pancreatitis [1].

## Conclusion

Pancreatic injury in blunt abdominal trauma in adolescent patients is infrequent and often difficult to diagnose. This case emphasises the need for clinical suspicion based on mechanism of injury, even in a relative innocuous collision during a sporting match. Patients who fail to improve over a period of observation with serial clinical examinations should be evaluated with further imaging.

Grade IV pancreatic injury can be effectively managed endoscopically and such injuries should be promptly referred to a specialist centre for appropriate and timely management.
